# A study on microstructural, mechanical properties, and optimization of wear behaviour of friction stir processed AZ31/TiC composites using response surface methodology

**DOI:** 10.1038/s41598-024-69348-w

**Published:** 2024-08-12

**Authors:** T. Satish Kumar, R. Raghu, G. Suganya Priyadharshini, Robert Čep, Kanak Kalita

**Affiliations:** 1 Department of Mechanical Engineering, Amrita School of Engineering, Amrita Vishwa Vidyapeetham, Coimbatore, 641112 India; 2 Department of Mechanical Engineering, Sri Ramakrishna Engineering College, Coimbatore, Tamil Nadu India; 3 Department of Mechanical Engineering, Coimbatore Institute of Technology, Coimbatore, India; 4 Department of Machining, Assembly and Engineering Metrology, Faculty of Mechanical Engineering, VSB-Technical University of Ostrava, 70800 Ostrava, Czech Republic; 5 Department of Mechanical Engineering, Vel Tech Rangarajan Dr. Sagunthala R&D Institute of Science and Technology, Avadi, 600062 India; 6 University Centre for Research & Development, Chandigarh University, Mohali, 140413 India

**Keywords:** AZ31 alloy, TiC particles, Friction stir processing, Microstructure, Hardness, Tensile properties, Wear optimization, Mechanical engineering, Materials science

## Abstract

The primary objective of this study is to investigate the microstructural, mechanical, and wear behaviour of AZ31/TiC surface composites fabricated through friction stir processing (FSP). TiC particles are reinforced onto the surface of AZ31 magnesium alloy to enhance its mechanical properties for demanding industrial applications. The FSP technique is employed to achieve a uniform dispersion of TiC particles and grain refinement in the surface composite. Microstructural characterization, mechanical testing (hardness and tensile strength), and wear behaviour evaluation under different operating conditions are performed. Response surface methodology (RSM) is utilized to optimize the wear rate by considering the effects of process parameters. The results reveal a significant improvement in hardness (41.3%) and tensile strength (39.1%) of the FSP-TiC composite compared to the base alloy, attributed to the refined grain structure (6–10 μm) and uniform distribution of TiC particles. The proposed regression model accurately predicts the wear rate, with a confirmation test validating an error percentage within ± 4%. Worn surface analysis elucidates the wear mechanisms, such as shallow grooves, delamination, and oxide layer formation, influenced by the applied load, sliding distance, and sliding velocity. The enhanced mechanical properties and wear resistance are attributed to the synergistic effects of grain refinement, particle-accelerated nucleation, the barrier effect of TiC particles, and improved interfacial bonding achieved through FSP. The optimized FSP-TiC composites exhibit potential for applications in industries demanding high strength, hardness, and wear resistance.

## Introduction

Magnesium alloys gained significant attention in the aerospace and automotive sectors owing to their specific strength, low density, superior machinability, and excellent castability^1,2^. However, the inherent low strength, poor ductility, and limited cold workability of magnesium alloys have restricted their widespread applications^3–5^. To overcome these limitations, researchers have explored various strategies, including grain refinement and reinforcement with ceramic particles or fibres, to improve the mechanical performance of magnesium alloys^6,7^. Friction stir processing (FSP) has become a promising solid-state processing technique available for creating surface composites and fine-tuning the grain structure of magnesium alloys^8,9^. FSP is a severe plastic deformation technique derived from friction stir welding, which involves plunging a rotating tool into material and traversing it along the desired path. During this process, the material experiences intense plastic deformation and frictional heating, leading to dynamic recrystallization and a refined microstructure^10,11^. Additionally, FSP enables uniform dispersion of reinforcement particles within the matrix, resulting in the formation of surface composites with enhanced mechanical properties^12,13^.

Numerous studies have been conducted to investigate the effects of various reinforcement particles on the microstructure and mechanical performance of magnesium alloys processed via FSP. AZ31 magnesium alloys processed via Friction Stir Processing (FSP) exhibit enhanced mechanical properties and refined microstructures. FSP followed by rolling results in increased ductility and strength, with AZ31/GNP composites showing an ultimate tensile strength of 296 MPa and an elongation of 15.7% due to grain refinement and second phase homogenization^14^. Additionally, FSP of AZ31 reinforced with MoS_2_ improves tribological behavior, hardness, and corrosion resistance, making it suitable for various applications^15^. Furthermore, Friction Stir-Surface Mechanical Attrition Treatment (FS-SMAT) refines grain size and enhances micro-hardness in AZ31 magnesium alloys, with different effects observed based on the type of stir tool used^16^. Moreover, the incorporation of nano-TC4 in AZ31 composites through spark plasma sintering and hot extrusion increases yield strength, ultimate tensile strength, and failure strain due to grain refinement and interfacial bonding^17^. Overall, FSP techniques effectively optimize the microstructure and mechanical properties of AZ31 magnesium alloys, offering improved performance for various industrial applications^18^.

Titanium carbide (TiC) is a promising alternative reinforcement material owing to its higher hardness, better thermal stability, and good wear resistance^19,20^. Most of the existing studies on AZ31/TiC composites fabricated through FSP have focused primarily on the microstructural and mechanical characterization^21,22^. However, there is a lack of investigations on the wear behaviour and optimization of wear properties for these composites. Additionally, the influence of process parameters on the wear performance has not been extensively explored. This study aims to bridge these gaps by conducting a systematic evaluation of the wear behaviour and employing response surface methodology (RSM) to optimize the wear rate of the AZ31/TiC composite by considering the effects of process parameters. By doing so, this research provides valuable insights into the wear mechanisms and contributes to the development of wear-resistant AZ31/TiC composites for industrial applications.

In this context, the present study aims to investigate the microstructural, mechanical, and wear behaviour of AZ31/TiC surface composites through FSP. The effects of TiC reinforcement and FSP on the microstructure, hardness, and tensile properties of composites will be evaluated. Furthermore, the dry sliding wear behaviour of composites will be evaluated under various operating conditions using response surface methodology (RSM). RSM is a statistical technique that enables optimisation of process parameters by establishing mathematical models and evaluating the relationships between input variables and responses^23,24^. By employing RSM, the wear behaviour of the composites can be optimised, and the influential factors governing the wear behaviour can be identified.

## Materials and methods

**Figure 1:**
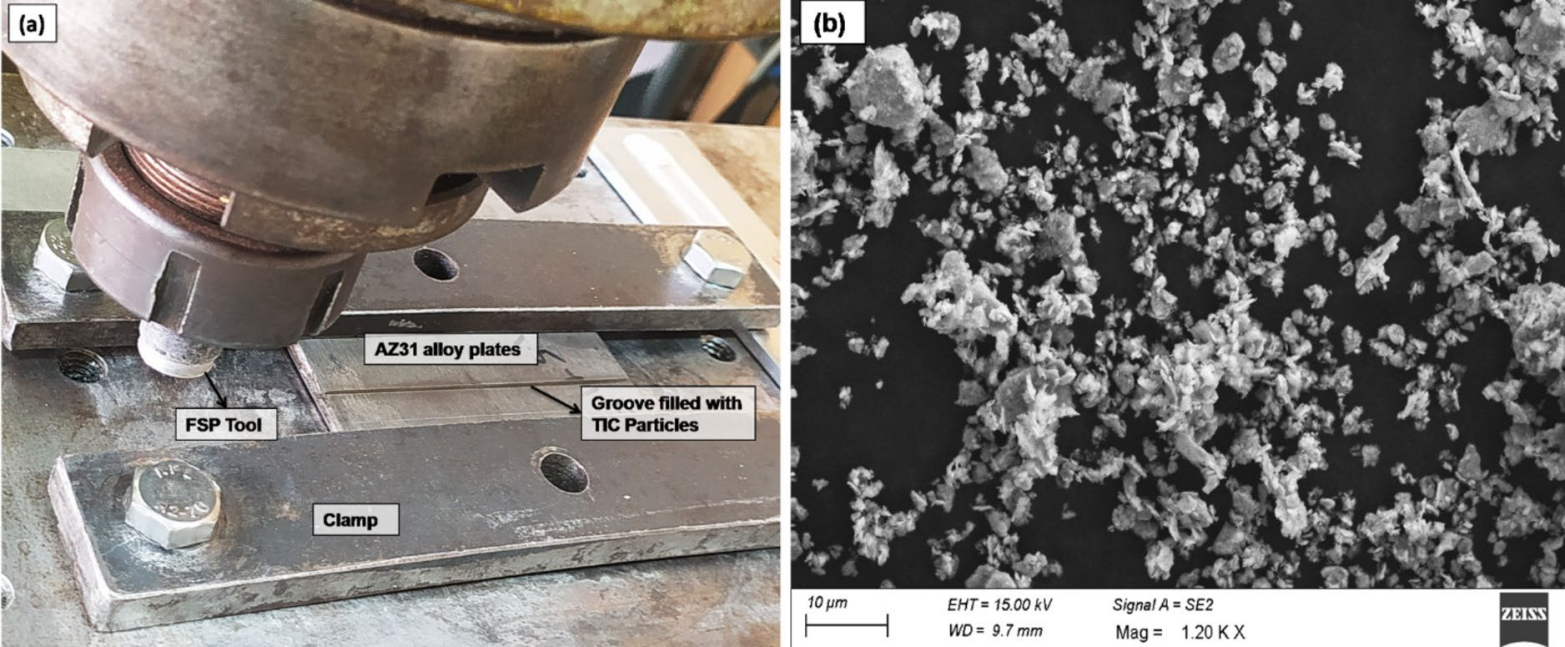
(a) Schematic view of FSP process (b) SEM image of the TiC particles.

The 100 × 100 × 6 mm commercially available AZ31 magnesium alloy is obtained and used as a base metal, and TiC particles (average size of 5 µm) are used as reinforcement. The volume percentage of TiC particles used in the fabrication of the AZ31/TiC composite was 15%. This percentage was determined based on preliminary experiments and literature studies, which suggested that a TiC content between 10 and 20% by volume could lead to significant improvements in mechanical properties and wear resistance while maintaining good composite integrity. The rationale behind selecting 15 vol% TiC was to strike a balance between achieving substantial mechanical property enhancements and maintaining good processability and composite integrity. Higher TiC content can lead to increased hardness and wear resistance but may also result in challenges during processing and potential composite embrittlement. For reinforcing 15 vol% TiC particles, a trapezoidal groove was machined on the AZ31 base metal surface with a width of 1.3 and 0.4 mm (top to bottom) and a depth of 4 mm. The calculated mass of TiC particles for 15 vol% reinforcement was carefully weighed and packed into the groove. The friction stir processing (FSP) tool was used to consolidate and disperse the TiC particles into the AZ31 matrix, forming the surface composite. Figure 1a shows the schematic view of FSP process and Fig. 1b shows the SEM image of the TiC particles used in the present study. Test samples are cleaned of oil and other contaminants using acetone. The TiC particles are compacted in the base metal groove using the pin less FSP tool. For FSP, a tungsten carbide cylindrical tapered profile pin tool with a 16 mm shoulder diameter, 4 mm length, and 4.5 pin diameters is utilized. For an efficient FSP, process parameters remain constant at 20 mm/min for traversal and 900 rpm for rotation with a 10 kN applied force. Following FSP, samples of the base alloy and its composite were collected for microstructural examination. Before etching with a mixture of 70 ml of ethanol, 4 ml of picric acid, and 10 ml of acetic acid, the surface composite samples were polished.

Microstructural investigations are conducted by a polarized light microscope (Carl Zeiss Axio Scope A1). The SEM (ZEISS, Model: Gemini 300) is used for scanning electron microscopy (SEM) and X-ray diffraction (XRD) to characterize TiC particles and processed samples. A Vickers Hardness tester with a 15 s dwell time, a 100-gf load, and adherence to ASTM 384 is used to perform the microhardness test. Tensile test samples' dimensions are sized in compliance with the ASTM E8 standard for treated zones transverse direction in accordance with ASTM G99^25,26^. Using pin-on-disc apparatus, a wear test is conducted in a room-temperature (33 ± 2 °C) dry sliding environment. The wire electric discharge machining was used to prepare pins of 5.0 × 5.0 × 10.0 mm. The EN31 steel used to make the disc has a hardness of around 65 HRC. Several grits of emery paper, up to 1200 grit, were used to polish the pin sample. To get the overall wear rate of the samples, the mean of the three measurements is taken as the final value. The design of the experiments serves as the basis for the wear test. The wear rate of AZ31-TiC FSP specimens is assessed using three components and five levels of process parameters. To assess the interfacial bonding and impact of TiC powder on base metal during wear processes, worn surfaces are examined. An empirical model is created to predict wear response, and RSM under CCD is used to find the optimal wear rate. The ANOVA method is often employed to examine the impact of process factors on wear rate.

## Results and discussion

### Microstructural studies

**Figure 2:**
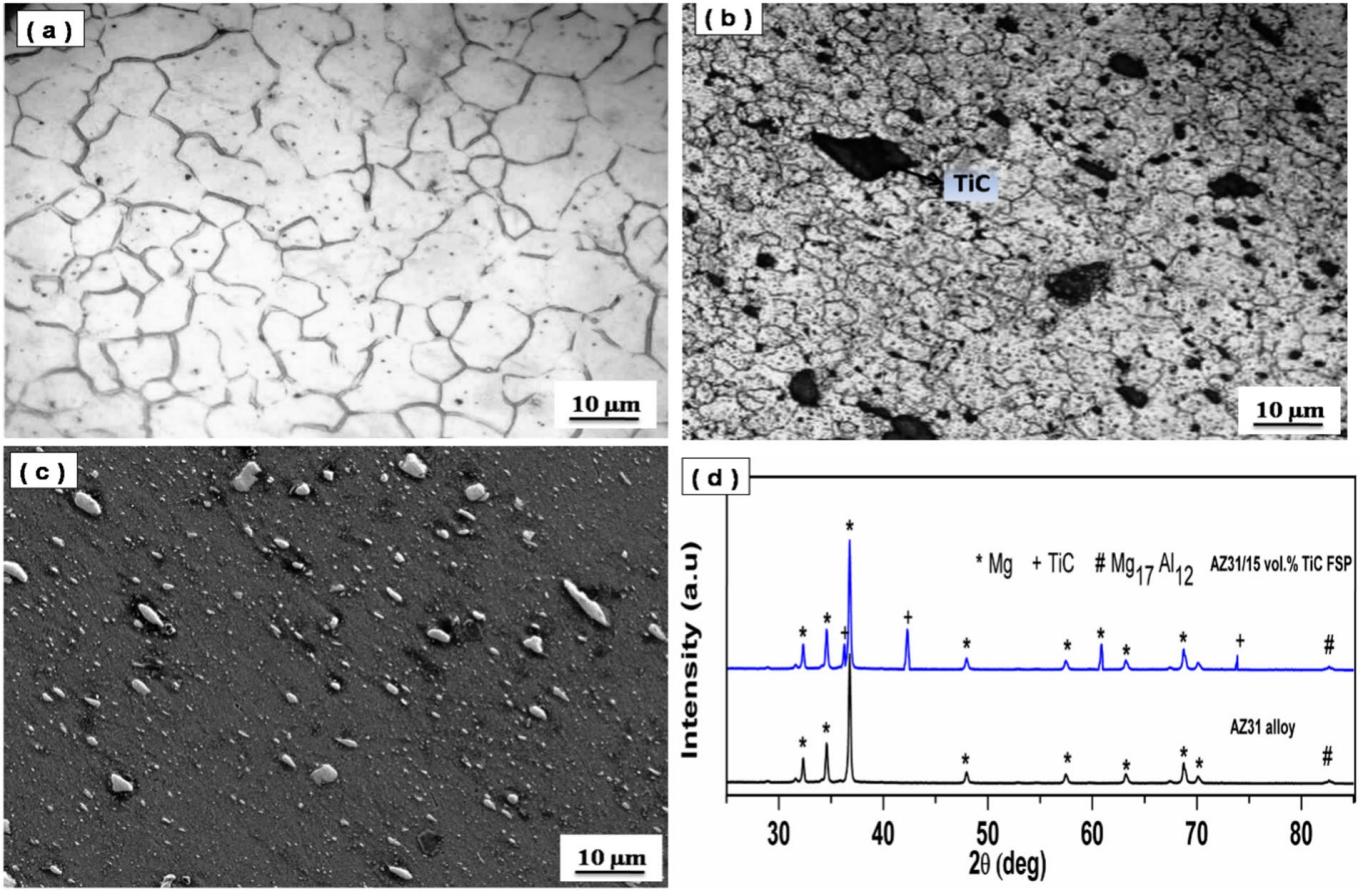
(a) FSP AZ31 alloy microstructure (b) AZ31-15 vol.% TiC FSP composite microstructure, (c) SEM image of AZ31-15 vol.% TiC FSP composite and (d) XRD pattern for both FSP AZ31alloy and AZ31-15 vol.% TiC FSP composite.

The FSP AZ31 alloy microstructure is shown in Fig. 2a, along with the microstructure of the AZ31-15 vol% TiC FSP composite Fig. 2b, the AZ31-15 vol% TiC FSP SEM image Fig. 2c, and the XRD pattern for the AZ31-15 vol% TiC FSP composite and the FSP AZ31 alloy Fig. 2d. Through a combination of tool rotation and traverse speed, the induced frictional TiC results in highly localized plastic deformation. Flaws such as onion rings, oxide layers, tunnelling cavities, and grooves are absent in the stir region of treated samples. Because there is enough frictional TiC on the defect-free surfaces, SPD may effectively use FSP to strengthen TiC particles on the base metal. Figure 2b illustrates the presence of uniformly distributed fine-equiaxed grains present in the TiC FSP-treated sample. Discontinuous dynamic recrystallization (DRX) refines the grains in the SZ, while adequate frictional heat refines the SPD. The FSP-15 vol% TiC sample displays uniformly dispersed grains with average grain sizes ranging from 6 to 10 µm.

In this study, micron-sized TiC particles (5 µm) were used as reinforcement for the fabrication of AZ31/TiC surface composites through friction stir processing (FSP). The effectiveness of particle size on grain refinement is a crucial factor in the development of metal matrix composites. Generally, nano-sized particles are considered more effective in inducing grain refinement compared to micron-sized particles. The underlying reasons for this are higher number density, increased particle–matrix interface areas and pinning effect. While nano-sized particles offer advantages in terms of grain refinement, their incorporation into metal matrices can present challenges related to agglomeration, clustering, and uniform dispersion. Improper dispersion of nano-sized particles may lead to non-uniform grain refinement and potential degradation of mechanical properties. In the present study, the use of micron-sized TiC particles (5 μm) has successfully resulted in significant grain refinement in the AZ31/TiC surface composite, with grain sizes ranging from 6 to 10 μm in the stir zone. This grain refinement can be attributed to the combined effects of discontinuous dynamic recrystallization (DRX) facilitated by the FSP process and the particle-accelerated nucleation (PAN) mechanism induced by the presence of TiC particles. Although nano-sized particles may offer improved grain refinement potential, the micron-sized TiC particles used in this study have proven effective in achieving substantial grain refinement and enhancing the mechanical properties and wear resistance of the AZ31/TiC surface composite.

The FSP-TiC sample's cross-sectional image (Fig. 2b) demonstrates the homogenous dispersion of TiC particles within the SZ. Because enough frictional heat is generated during FSP, the TiC components are uniformly distributed in all directions without segregation or grouped areas. The SEM micrograph (Fig. 1b) shows a strong bond between the TiC particle and the base metal. The XRD pattern (Fig. 2d) confirms the presence of TiC particles in the composite sample.

**Figure 3:**
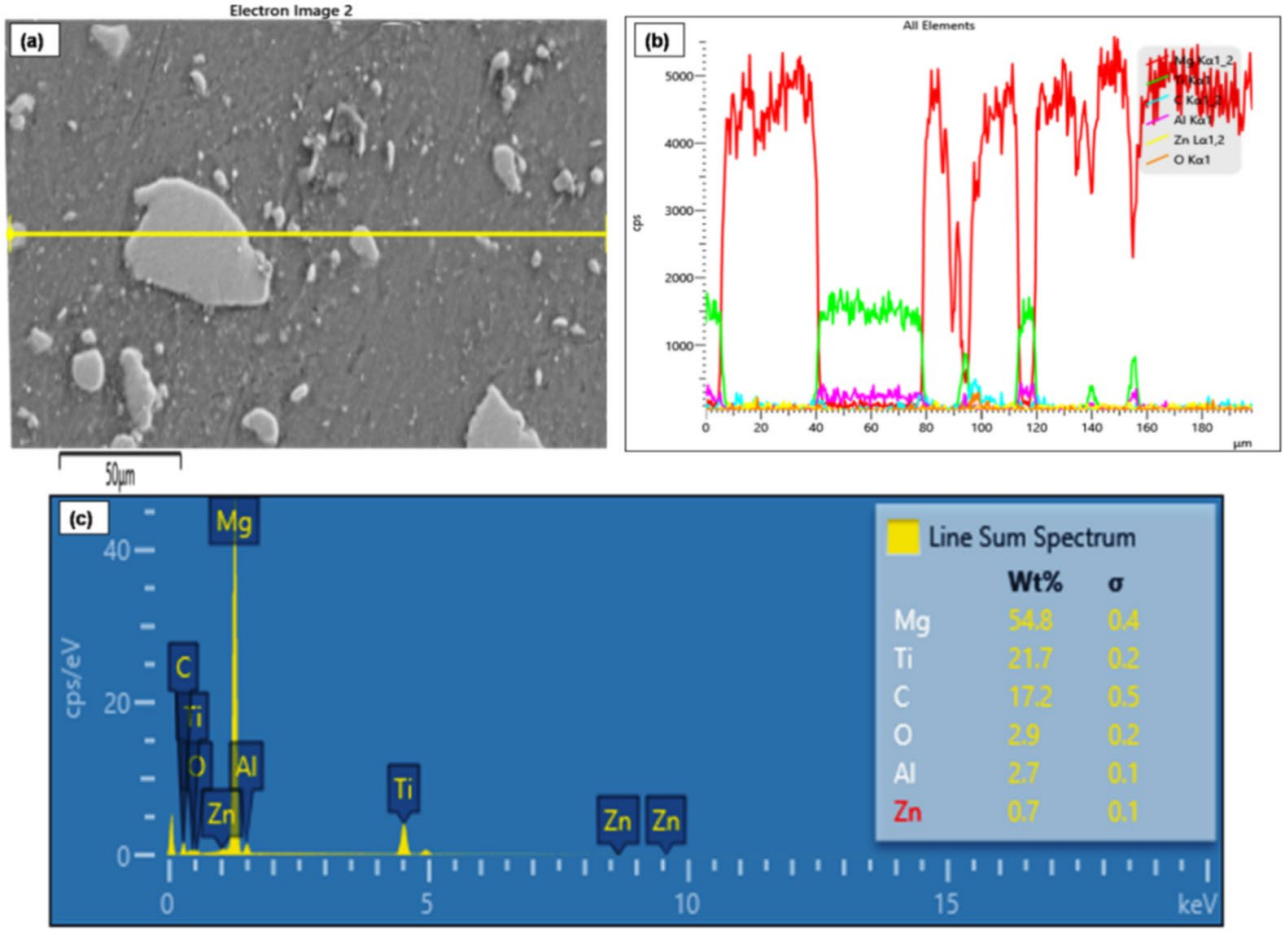
(a) Surface-line mapping of the AZ31-TiC FSP sample (b) Line mapping with element distribution and (c) EDS graph of the mapped region.

**Figure 4:**
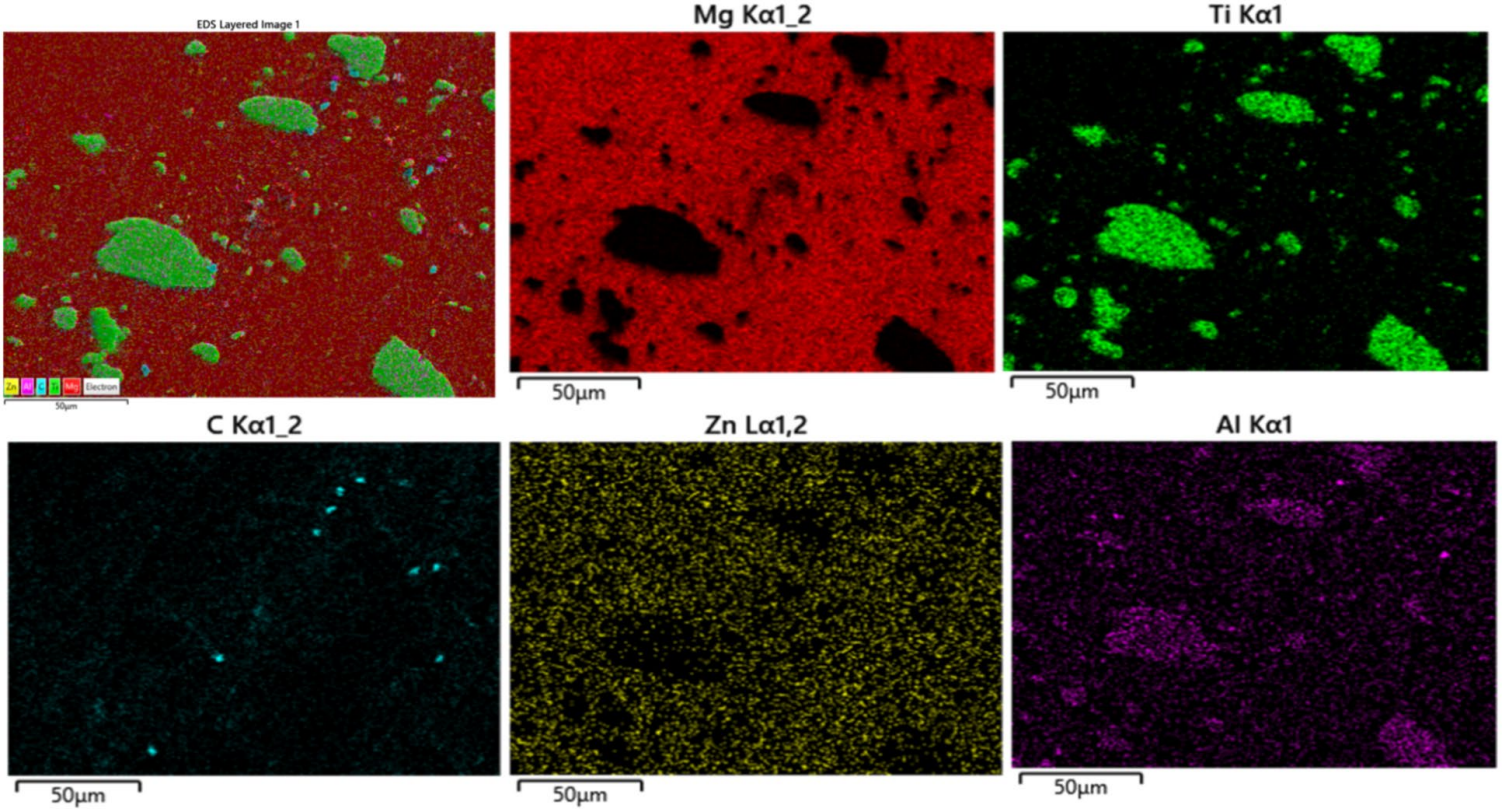
Distributions and mapping of TiC elements across the SZ of the AZ31-15 vol. % TiC FSP composite.

Line mapping analysis is used to identify the elements that are present in the FSP-TiC sample surface cross-section (Fig. 3a). The mapping indicates the presence of TiC particles in the stir zone region. Elements in each region and the line mapping are shown in Fig. 3b. The sharp change in the elemental line near the TiC particle and the AZ31 base metal indicates that the interface is clean and free from interfacial reactions. The EDS results in Fig. 3c further confirms the XRD results, indicating the presence of TiC particles. The enhanced TiC-base metal metallurgical bonding can also be attributed to the SPD^27^. The distribution and mapping of TiC elements across the SZ of the AZ31-15 vol. % TiC FSP composite is shown in Fig. 4. The distributions of individual elements indicate a strong interfacial connection between the TiC particles and the base metal. In Fig. 3, FSP AZ31 alloy microstructure shows coarse equiaxed grains with an average grain size of 50 µm.

DRX and SPD are demonstrated by grain expansion and refined grains on the SZ of the FSP-TiC sample. The solidification zone (SZ) of the sample contains TiC particles, which are preventing the grain size from increasing.

It was suggested that the quenching effect also lowered grain size through different thermal coefficients of expansion of TiC and base metals. Moreover, the dislocation density is elevated by the impact of TiC particles because of the variation in thermal expansion coefficients. Consequently, the particle-stimulated nucleation (PSN) mechanism successfully causes recovery kinetics^28^.

### Assessment of mechanical characteristics

**Figure 5:**
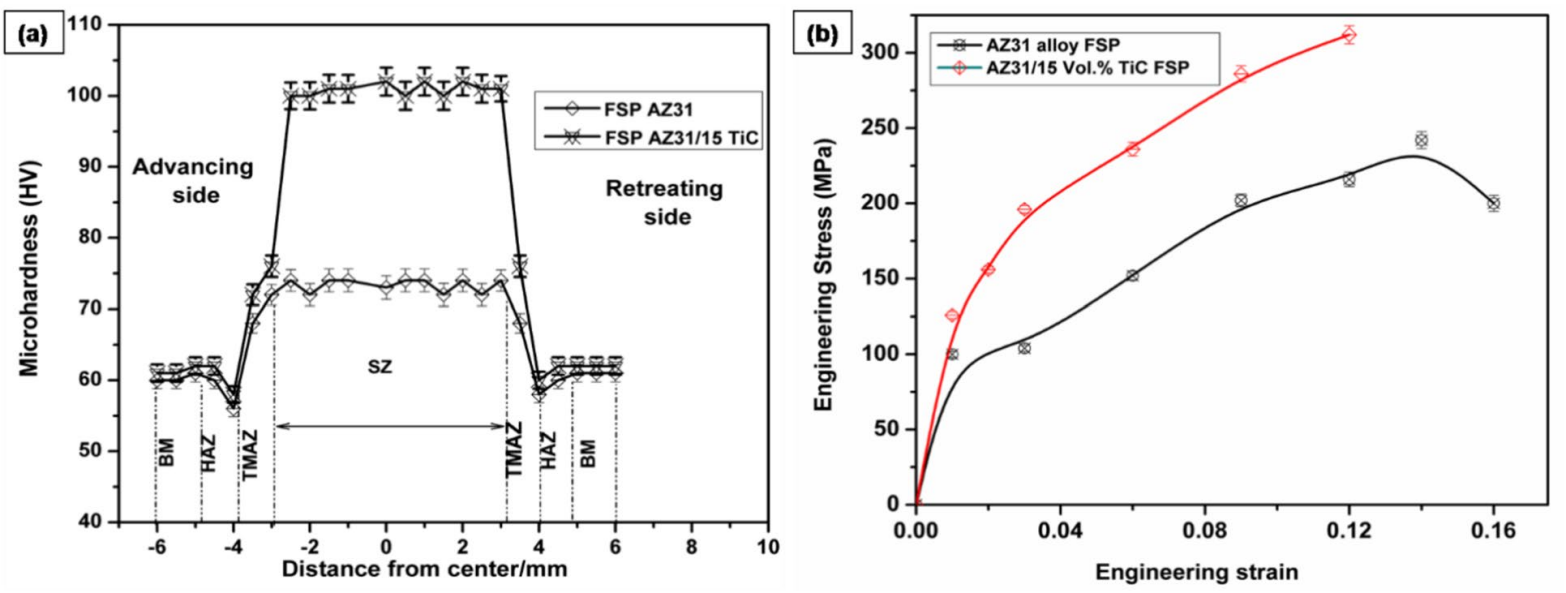
(a) Hardness distributions across SZ (b) Tensile strength of AZ31-15 vol. % TiC FSP composite.

The microhardness distribution profile of the FSP-base and FSP AZ31/15 vol.% TiC samples is displayed in Fig. 5a. Because FSP and the presence of TiC particles refine matrix grains, the FSP-TiC composite sample has increased microhardness. The FSP-AZ31/15 vol.% TiC sample has a higher microhardness of 104 HV at the centre of SZ, which is 67% higher than FSP-base metal 62 HV. Higher microhardness results from the TiC particles improved strengthening effects. Because of the materials’ softening from induced frictional TiC, the value of microhardness decreased around the SZ during FSP^29^.

The FSP TiC sample’s SZ has better microhardness due to the following characteristics: (i) the presence of TiC particles; (ii) the effect of quench hardening; and (iii) the Hall–Petch connection through grain refinement. Additionally, hardness is improved by the production of many sub-grains, a large fraction of HAGB over the base sample, a uniform distribution of TiC particles, and inhibition of grain expansion^30^. The rule of mixtures^31^ states that enhanced microhardness results from base metals supplemented with TiC particles. Quench hardening, which increases microhardness, is caused by a number of factors, including grain refinements, generated frictional heating during FSP, and the coefficient of heat differential between TiC particles and the base metal^32^. Furthermore, there is less fluctuation in the microhardness profile, indicating that refined grains in SZ are distributed uniformly^33^. The tensile characteristics of the FSP-TiC and FSP-base materials are assessed using prepared test samples that are obtained in a transverse direction. The tensile strength of the FSP-AZ31/15 vol% TiC sample is 294 MPa, which is 30% greater than that of the FSP-AZ31 alloy, which is 222 MPa (Fig. 5b). Grain refinements are responsible for the enhanced tensile capabilities of reinforced TIC particles^34^. Grain refinements in the SZ are achieved through the use of DRX, produced by friction heat generated between the tool and work material in the FSP. The processes of grain growth and finer grains are two important aspects of the recrystallization process.

Grain reduction is caused by the tool's constant stirring motion, which guides the creation of nucleation sites, while grain size increases are caused by friction heating. The tool's rotation speed greatly influences the distribution and fineness of grains. The material’s resistance to deformation is strengthened by the SZs refined granules^35^. The localized microstructure alterations and discontinuous DRX associated with the FSP-AZ31/15 vol% TiC samples strengthened refined grains^36^. Because of the difference in the base metals and reinforcement's thermal expansion coefficients, the FSP-AZ31/15 vol% TiC samples dislocation strengthening increased. The inclusion of TiC particles may obstruct dislocation migration, leading to even higher tensile strength. In the tensile test, the stress concentration was effectively decreased by the TiC particles’ spherical shape, which is devoid of sharp edges and uneven profiles^37^. Higher tensile characteristics are also a result of stacking faults, planner defects, and FSP-assisted grain size reduction in treated samples, according to the Hall–Petch relation^38^.

### FSP-TiC sample wear optimization

Higher microhardness results from smaller grain sizes that stop dislocation motion during deformation. FSP-AZ31/15 vol% TiC samples' wear resistance can be enhanced by improved grain boundary area, homogeneous distribution, interfacial bonding of TiC particles, and resistance to abrasive wear by fine grains^39^.

Additionally, Archard's rule^40^ was used to calculate the grain reduction and wear resistance of refined grains. As a result, a wear test was conducted, and parameters were improved using Archard's law and the literature. Table 1 displays the configurable wear parameters together with their respective levels. Table 2 lists the three components and five wear parameter levels that result in 20 runs of tests through RSM. The CCD module in RSM conducts 20 runs of experiments with appropriate levels of process parameters based on the Design of Experiments (DOE). The weight loss of the FSP-TiC sample is used to compute the volumetric wear rate.

**Table 1:** Coded and actual value of parameters and their levels.

Independent factors	Notations	− 2	− 1	0	+ 1	+ 2
Applied load (N)	A	10	18	30	42	50
Sliding distance(m)	B	500	905	1500	2095	2500
Sliding velocity (m/s)	C	0.5	0.9	1.5	2.1	2.5

**Table 2:** Wear experiment results.

Run order	Applied load (N)	Sliding distance(m)	Sliding velocity (m/s)	Volumetric wear rate × 10^–3^
1	30	1500	2	2.505
2	18	905	2	2.288
3	18	2095	1	2.389
4	42	2095	1	2.852
5	30	1500	1	2.388
6	30	1500	2	2.387
7	42	905	1	2.553
8	30	1500	2	2.388
9	18	2095	2	2.342
10	30	1500	3	2.452
11	42	2095	2	2.789
12	30	2500	2	2.556
13	30	1500	2	2.387
14	30	1500	2	2.388
15	30	1500	2	2.389
16	10	1500	2	2.488
17	42	905	2	2.539
18	50	1500	2	2.785
19	18	905	1	2.368
20	30	500	2	2.506

The volumetric wear rate is determined using Eq. 1^41^. The predicted wear rate is shown in Table 3.

1 $$ {\text{Volumetric wear rate = loss in weight (sliding distance) }} $$

**Table 3:** Confirmation test results.

Run order	Applied load (N)	Sliding distance (m)	Sliding velocity (m/s)	Predicted values	Experimental values	Error %	Status
1	20	1250	2.3	2.000	2.065	3.25	Significant
2	35	2000	1.8	2.096	2.125	1.38	
3	40	1500	0.7	2.305	2.345	1.73	

**Figure 6:**
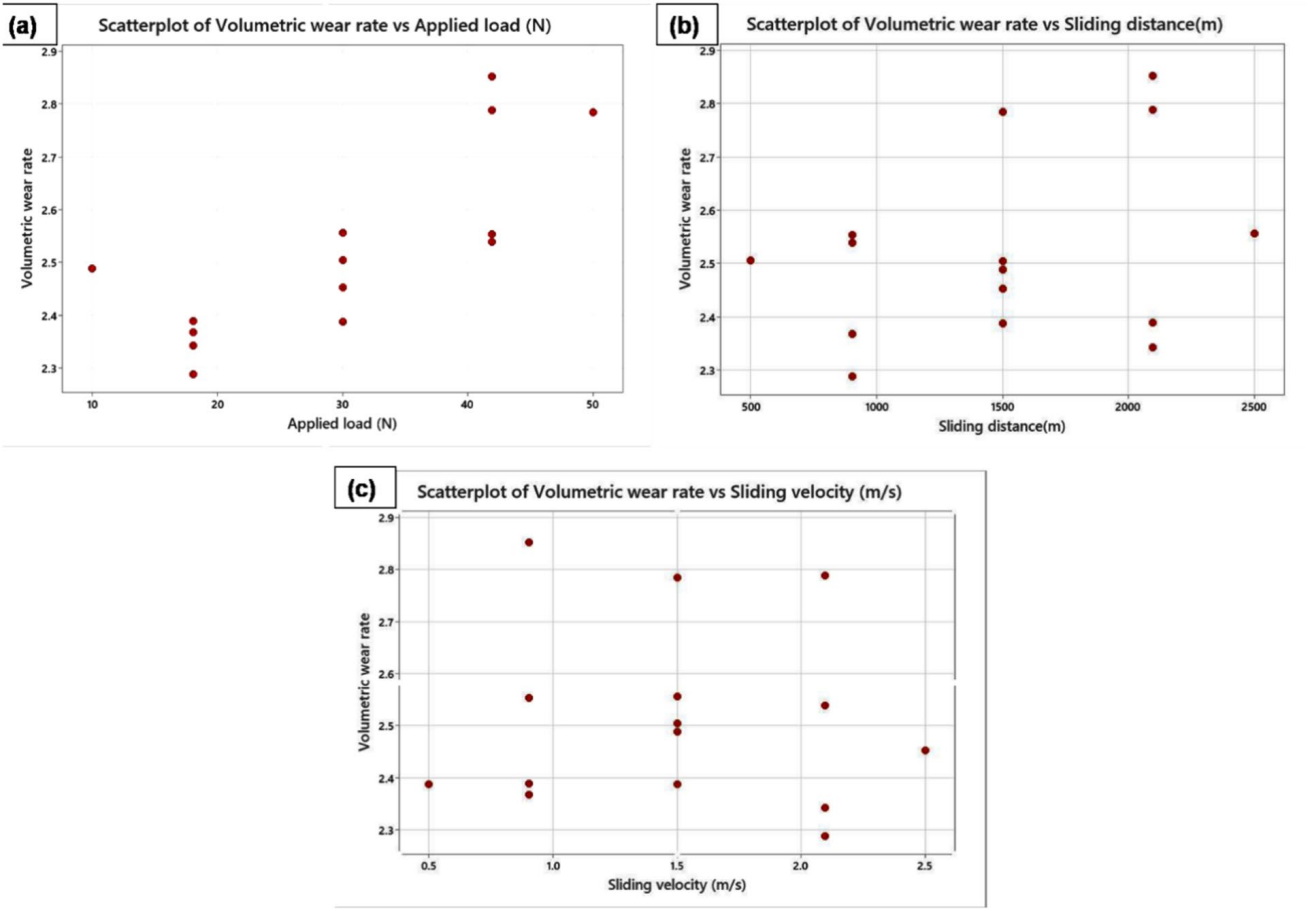
Plots of scatter for (a) load applied (b) distance of sliding (c) sliding velocity.

At 95.41% and 94.89%, respectively, the adjusted R^2^ and R^2^ values are extremely close to one another. The developed model is significant at a 95% confidence level. Equation 2 is the wear rate regression model that was obtained. Based on input process parameters, regression modelling is used to calculate the wear rate of the FSP-TiC sample. A confirmation test is utilised to confirm the wear rate and accuracy of the constructed model. Several levels of process parameters that were left out of the initial design serve as the basis for the confirmation analysis. Confirmation test results were tabulated in Table 3. Using DOE, anticipated and experimental wear rates are used to compute the error percentile. The experiment's error percentile is within ± 4%. As a result, the built-in regression model showed that it was possible to accurately forecast the wear rate. Regression models and the CCD module are used in a number of research projects to predict the answers^42^. On both sides of the slope, the scatter diagram (Fig. 6a–c) clearly illustrates the relationship between residuals and process parameters.

**Figure 7:**
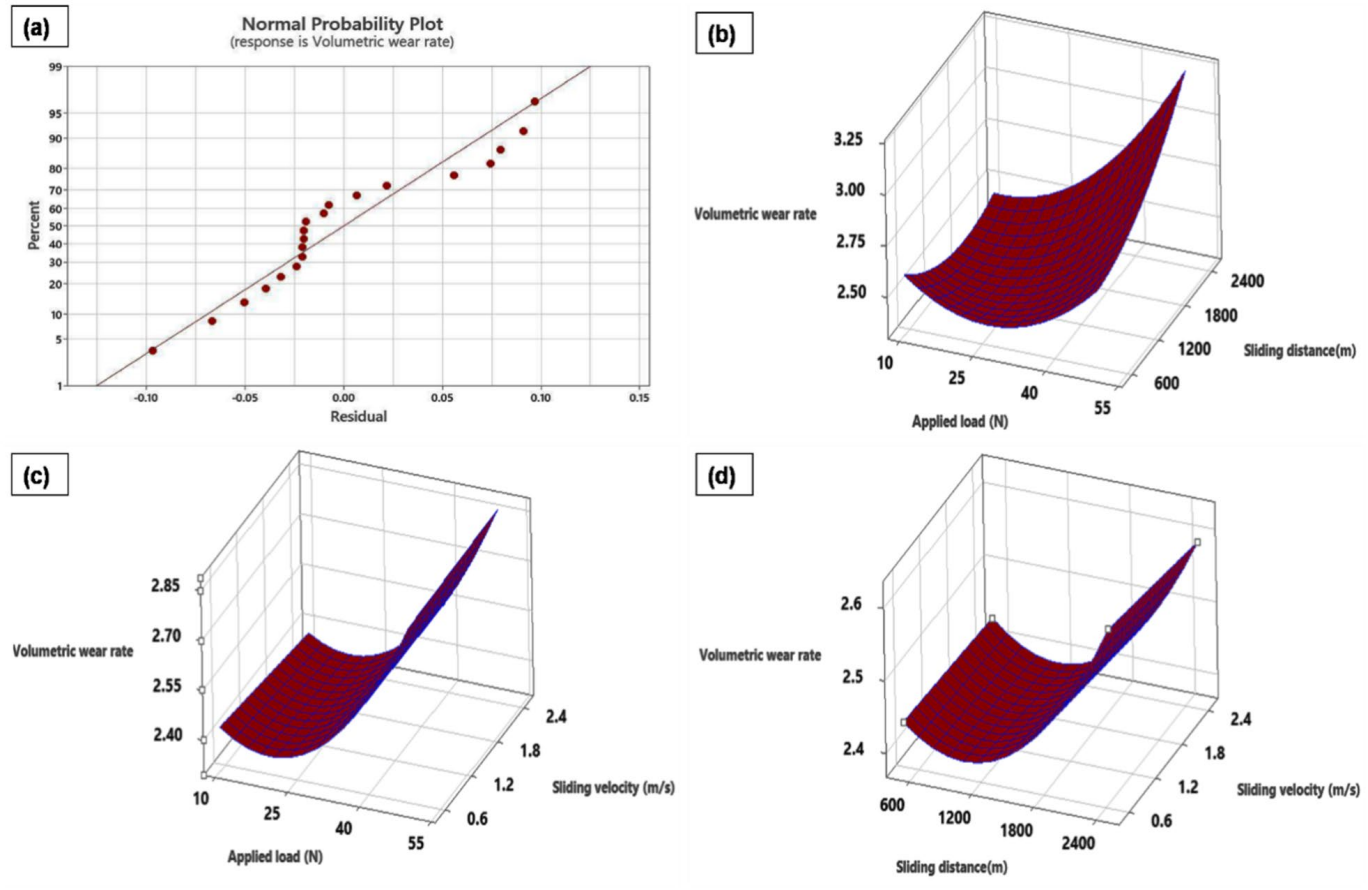
(a) Correlation between the expected and real values. Wear rate in a 3D surface plot (b). sliding distance vs applied load, (c) sliding velocity vs applied load, (d) sliding distance vs sliding velocity.

The wear rate that is statistically significant for a given set of wear characteristics for processed samples that have undergone ANVOA analysis using RSM. Table 4 displays the wear rate results of the ANOVA study. For the chosen process parameters, a *p*-value (less than 5% probability value) is produced when 95% significance is shown for each term in the constructed models. For a particular level of process parameters, the wear rate models in use are substantial and helpful. The interplay between the expected and actual wear rate values is depicted in Fig. 7a through anormal probability plot.

2 $$ \begin{aligned} & {\text{Volumetric wear rate}} = {3}.0{94} - 0.0{35}0{\text{Applied load}}\left( {\text{N}} \right) = 0.000{\text{488 Sliding distance}}\left( {\text{m}} \right) \\ & \quad - \;0.0{\text{29 Sliding velocity}}\left( {{\text{m}}/{\text{s}}} \right) + 0.000{541}\;{\text{Applied load}}\left( {\text{N}} \right)*{\text{Applied load }}\left( {\text{N}} \right) \\ & \quad - \;0.000{\text{2 Sliding velocity }}\left( {{\text{m}}/{\text{s}}} \right)*{\text{Sliding velocity }}\left( {{\text{m}}/{\text{s}}} \right) \\ & \quad + \, 0.00000{\text{8 Applied load}}\left( {\text{N}} \right)*{\text{Sliding distance}}\left( {\text{m}} \right) + 0.000{88 } \\ & \quad {\text{Applied load}}\left( {\text{N}} \right)*{\text{sliding velocity }}\left( {{\text{m}}/{\text{s}}} \right) \\ & \quad - 0.00000{\text{6 Sliding distance }}\left( {\text{m}} \right)*{\text{sliding velocity }}\left( {{\text{m}}/{\text{s}}} \right) \\ \end{aligned} $$

**Table 4:** ANOVA evaluation for volumetric wear rate.

Source	DF	Seq. SS	Contribution (%)	Adj. SS	Adj. MS	F-value	*P*-value
Model	9	0.4156	88.36	0.4156	0.0462	8.44	0.001
Linear	3	0.2868	60.97	0.2868	0.0956	17.46	0.000
Applied load (N)	1	0.2494	53.02	0.2494	0.2494	45.56	0.000
Sliding distance (m)	1	0.0367	7.81	0.0367	0.0367	6.71	0.027
Sliding velocity (m/s)	1	0.0007	0.14	0.0007	0.0007	0.12	0.732
Square	3	0.1004	21.35	0.1004	0.0335	6.12	0.012
Applied load (N) × Applied load (N)	1	0.0781	16.60	0.0843	0.0843	15.40	0.003
Sliding distance(m) × Sliding distance (m)	1	0.0223	4.75	0.0221	0.0221	4.04	0.072
Sliding velocity (m/s) × Sliding velocity (m/s)	1	0.0000	0.00	0.0000	0.0000	0.00	0.997
2-Way Interaction	3	0.0284	6.04	0.0284	0.0095	1.73	0.224
Applied load (N) × Sliding distance (m)	1	0.0281	5.97	0.0281	0.0281	5.13	0.047
Applied load (N) × Sliding velocity (m/s)	1	0.0003	0.07	0.0003	0.0003	0.06	0.816
Sliding distance(m) × Sliding velocity (m/s)	1	0.0000	0.01	0.0000	0.0000	0.01	0.941
Error	10	0.0547	11.64	0.0547	0.0055		
Lack-of-fit	5	0.0433	9.20	0.0433	0.0087	3.78	0.085
Pure error	5	0.0114	2.43	0.0114	0.0023		
Total	19	0.4704	100.00

The wear rate interaction effect of process parameters is displayed in a 3D surface plot (Fig. 7b–d). Figure 7b shows the relationship between sliding distance, wear rate, and applied force. With each increase in sliding distance and applied force, the wear rate rises linearly. A lever that is applied forces the pin and counter plate into contact. A pin's contact with the counter plate rises with a load increment of 10–50 N, and this contact also lasts longer when sliding distance is added^43^.

The temperature at the sample-counter plate contact rises with increasing load and sliding distance, leading to a rising wear rate for FSP-TiC samples. Early research on wear behaviours showed similar results^44,45^. As demonstrated in Fig. 7c, the wear rate did, however, decrease with increasing applied load and sliding velocity augmentation. The sample makes more contact with the counter plate as a result of the reduced process parameter velocity. There is a large loss of material and a high wear rate when metals disengage from sample surfaces. The temperature rise at contact caused by sliding velocity results in an oxide layer on the pin surface. Even at higher loads, the oxide layer shields the specimen from adhesive wear^46^. The relationship between the FSP-TiC samples’ sliding velocity and sliding distance is seen in Fig. 7d. The sample's wear rate decreases as the sliding velocity increases. Even when the specimen comes into contact with a counter plate for an extended period of time (high sliding distance), it is protected from harm by an oxide layer that accumulates on the pin surface. Therefore, when sliding distance increases, wear rate experiences a minimum influence. Similar results regarding the wear process during experimental work were achieved by a pertinent investigation^47^. Because of their improved metallurgical bonding, dynamical recrystallization, and polished grains, the FSP-TiC samples show superior wear resistance.

The wear rate may have been lowered by the reinforced TiC particles, preventing the base metal from making direct contact with them. No TiC particles have peeled off, and their surface is devoid of cracks, which improves their resilience to wear. These characteristics demonstrate that the action of TiC particles on the base metal results in enhanced metallurgical bonding. Furthermore, the load-bearing ability of TiC particles transfers the load from the matrix to the reinforced material. By using a direct surface modification approach, the refined grains of the FSP-TiC samples result in better surface microstructural characteristics. The changed phase structure through FSP is the reason for the increased wear resistance of the FSP-TiC samples^48^. The FSP-TiC samples microhardness and wear rate display a strong connection, as demonstrated by the Hall–Petch relation and Archard law. Wear resistance may be improved by combining the efforts of micro-abrasion and micro-grain refining on a finer scale^49^. Because strengthened TiC particles serve as nucleation sites for fresh grain formation, the PSN mechanism increases wear resistance. Nucleation sites change the microstructure, improving its characteristics. The wear resistance is improved by the following properties: (i). strengthening of grain boundaries through the creation of new and refined grains; (ii). dislocation movement hindrance; (iii). creation of a new phase or fortification of an already-existing phase; and (iv). uniform dispersion of TiC particles in motion. Achieving the best possible wear resistance is largely dependent on the PSN mechanism^50^.

### Examination of worn surfaces of FSP-AZ31-TiC samples

**Figure 8:**
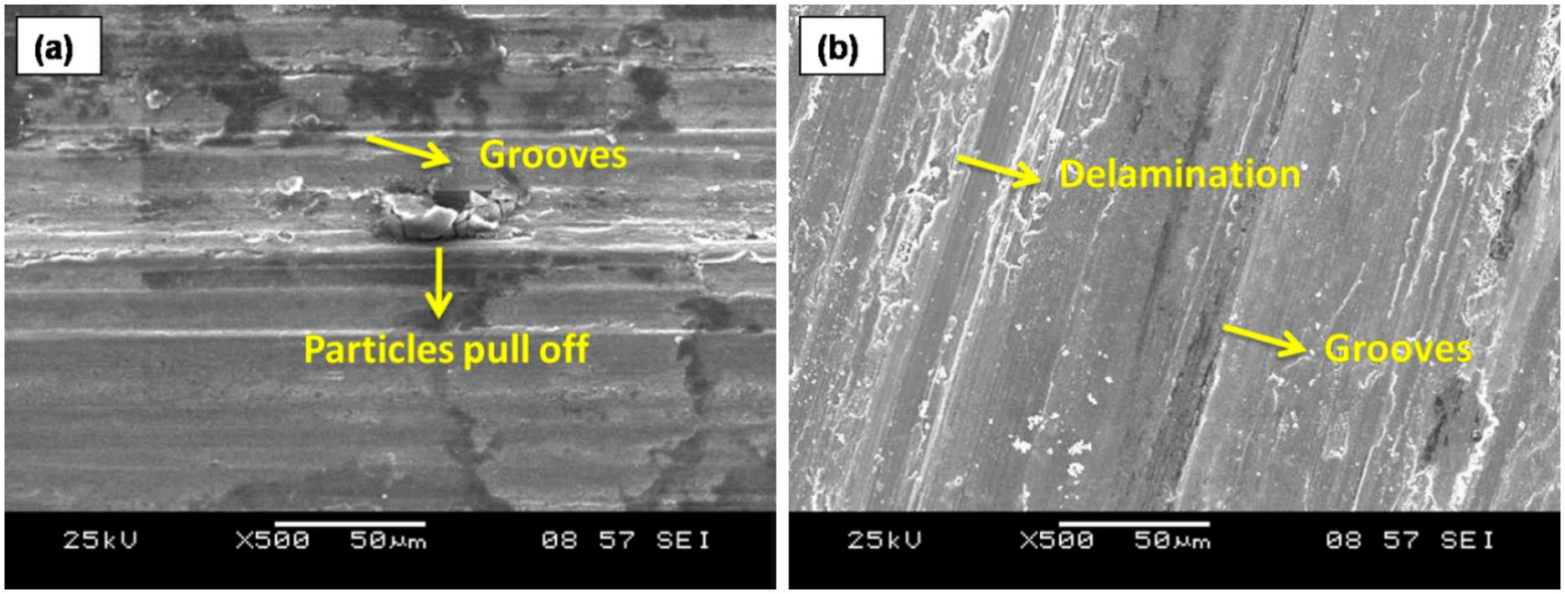
FSP AZ31/ 15 vol% TiC composite worn surfaces at an applied load of (a) 10 N and (b) 50 N.

The worn surface of FSP AZ31-TiC samples at various applied load levels is displayed in Fig. 8. Particles were pulled off of the worn surface in specific locations with shallow grooves at 10 N stress settings, as seen in Fig. 8a. The lowest frictional temperature that is reached with a 10 N load leads to a lower wear rate. There are a few shallow grooves on the worn surface owing to slight plastic deformation that lowers dislocation density. As the applied load increases, so does the dislocation density. The applied load is transferred from the base metal to TiC particles, which serve as the load-bearing components. As a result of an increased load, Fig. 8b displays delamination and continuous grooves in the sliding direction. The gradual application of load to the lever attachment is what causes the close contact between the pin and counter plate. The temperature that is produced at the interface between a specimen and a counter plate increases the material removal rate of FSP-TiC samples. Thus, at an applied force of 50 N, the sample wear rate increases. When a higher load (30–50 N) is applied, friction between the pin and counter plate increases the amount of heat created.

The high frictional heat caused by the deformation of worn surfaces results in increased material loss. As a result, it causes greater material delamination and persistent grooves on worn surfaces. The 3D surface plate of Fig. 7b confirms the greater wear rate caused by the increase in applied load. Trends in worn surface morphology are similar in nature^50^.

**Figure 9:**
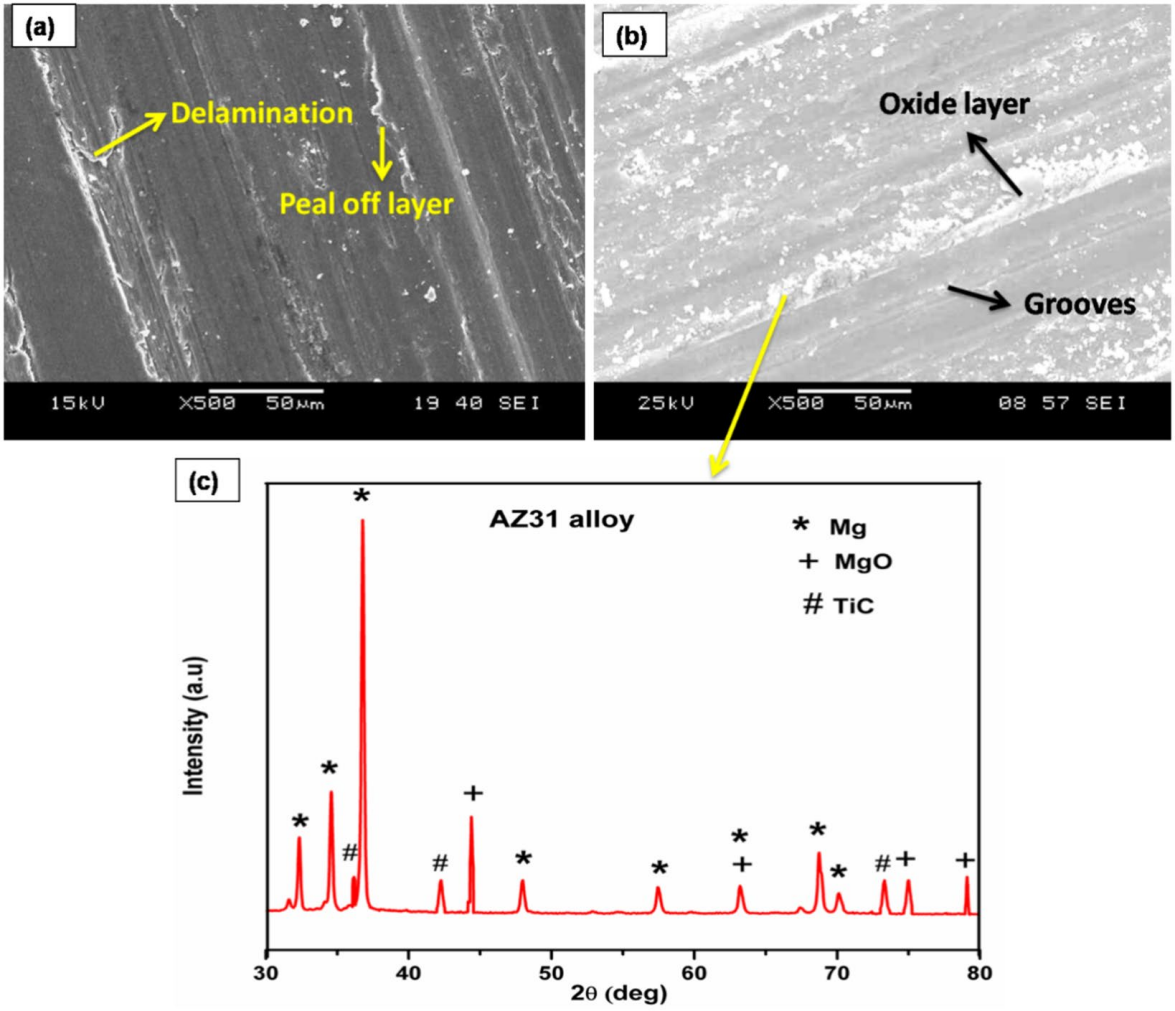
Worn surfaces with sliding distance (a) 500 m, (b) 2500 m and (c) XRD results of the worn surfaces.

The FSP-TiC sample experiences a wear rate increase as sliding distance rises because of longer pin and counter plate contact lengths. Contact stress that develops between the pin and counter plate causes a peel-off layer to form and delamination to be visible on the worn surface at a 500 m sliding distance (Fig. 9a). On worn surfaces, there is increased delamination because of the uneven dislocation. A high degree of delamination causes more materials to separate from the worn surfaces, which increases the wear rate. The worn sample is softened by the warmth produced by the SPD. The 2500 m sliding distance significantly lengthens the counter plate and pin contact period (Fig. 9b). Over an extended period of time, the temperature rises owing to increased contact between the pin and counter plate. Detachable materials glide over the specimen surface on a counter plate that is rolled up on the surface due to the counter plate's continual rotation. Eventually, materials detach from the surface of a specimen as a result of the counter plate rotating continuously to create continuous grooves on the sample surfaces.

**Figure 10:**
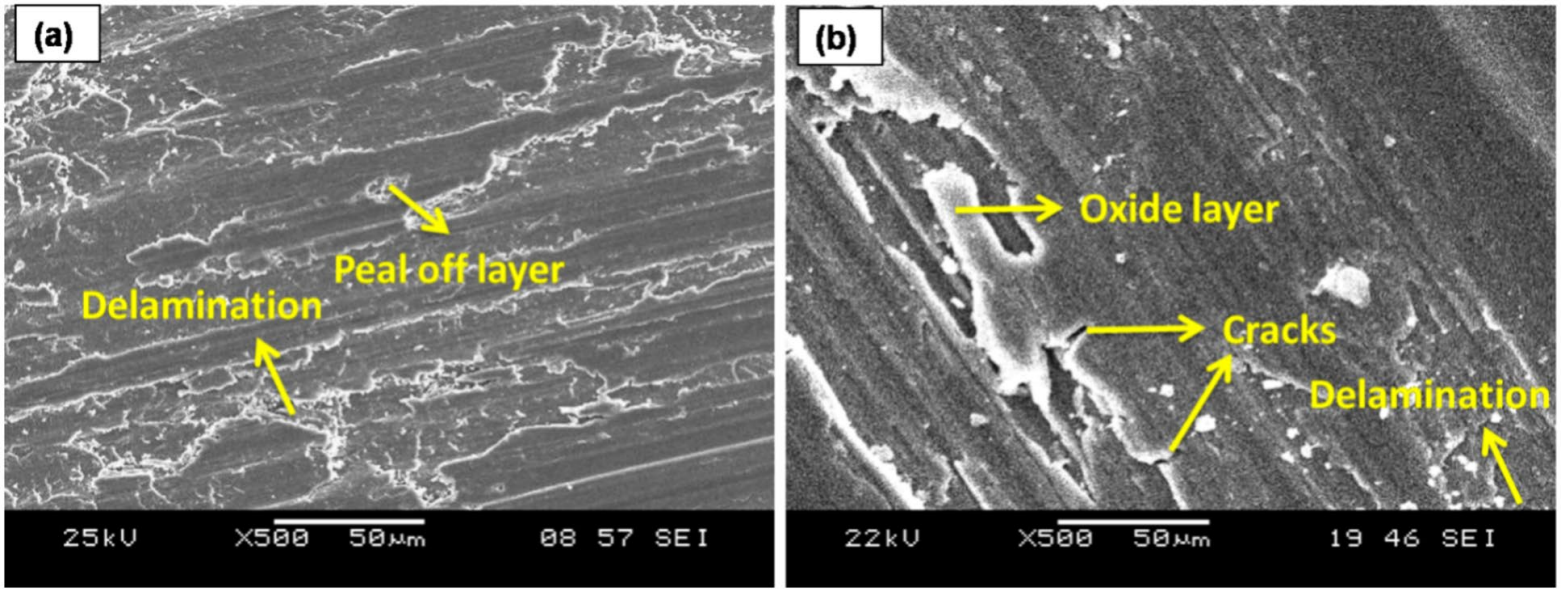
AZ31/ 15 Vol.% TiC composite sample's worn surfaces at sliding speeds of (a) 0.5 m/s and (b) 2.5 m/s.

Using the XRD examination of worn surfaces under sliding conditions, the elemental composition is evaluated (Fig. 9c). The results confirm the presence of an MgO layer on the worn surface. The oxide layer forms because the detached particles roll up and become softer due to the frictional temperature that is created. This process causes the surface to become resistant to wear. A 3D surface map is depicted in Fig. 7c is used to verify the sliding distance wear mechanism, which shows a similar pattern. Figure 10 shows a worn surface beneath an increase in sliding velocity. At sliding velocities of 0.5 m/s, peel-off materials and persistent delamination are observed (Fig. 10a). Because of the counter plate's initial velocity, a pin contacts it more thoroughly. At 0.5, the pin and counter plate's contact duration is getting longer. There is increased friction between the pin and counter plate as a result of the higher temperature. At a sliding velocity of 0.5 m/s, peel-off and delamination of materials result in greater material losses and suggest a higher sample wear rate.

Oxide layer growth on the pin surface reduces wear rate at a sliding velocity of 2.5 m/s (Fig. 10b). The sample has less direct contact with the counter plate due to the oxide layer produced, which shields the worn surfaces from damage. Therefore, the oxide layer prevents the separated metal from leaving the surface, resulting in a lower wear rate.

Overall, the key dominating wear mechanisms observed in the AZ31/TiC composite are (i) Abrasive wear due to the presence of hard TiC particles and debris ploughing/cutting the composite surface, (ii) Adhesive wear at higher applied loads and sliding distances, characterized by material transfer, junction formation, and subsequent material loss leading to grooves and delamination (iii) Oxidative wear with the formation of a protective MgO oxide layer, especially at higher sliding velocities, acting as a solid lubricant and reducing direct metal-to-metal contact and (iv) Delamination wear involving the detachment of thin layers or platelets from the composite surface, influenced by high loads, sliding distances, and the propagation of subsurface cracks.

Among the various key dominant mechanisms, abrasive wear is observed as the most dominant mechanism due to various factors such as (i) the hard ceramic TiC particles embedded in the AZ31 matrix can act as abrasive elements, causing material removal from both the composite surface and the counter face (steel disc) during sliding wear (ii) The detached TiC particles or debris generated during the wear process can further contribute to abrasive wear by ploughing and cutting the composite surface and (iii) The protrusion of TiC particles from the composite surface can enhance the abrasive action, leading to more material removal and wear. While other mechanisms like adhesive wear, oxidative wear, and delamination wear were also observed, the inherent hardness of the TiC particles and their potential to act as abrasive elements make abrasive wear the most dominant mechanism in this composite system.

As a result, wear rate decreases as sliding velocity increases, as the 3D surface map confirms (Fig. 7d). Because of the reinforced TiC particles' superior material flow, the base metal is distributed evenly, boosting interfacial bonding and enhancing wear resistance. By improving strength, the barrier effect of TiC particles on grain formation results in a minimum wear rate ^46–50^. The dislocation movements that are reduced by the impeding impact of the TiC particles lower the wear rate of the FSP-TiC sample. The wear rate of the FSP-TiC sample rises with each increase in applied load. However, during the wear analysis, there was a minor slowdown in wear rate owing to the high strength and hardness of the TiC particles reinforced on base metal, which limited material deformation. Improved wear resistance is facilitated by characteristics such as dynamic recrystallization, fine-grained separation by FSP, grain reduction, efficient TiC particle movement, the TiC barrier effect, and superior interfacial bonding^31^.

## Conclusions

The TiC particles are efficiently reinforced by the FSP. Evaluations are conducted on the effects of TiC particles on the microstructural, mechanical, and wear properties of the base metal. RSM analyses the wear rate optimisation and important factors, and the following conclusion is reached:

1. Through enhanced metallurgical bonding and homogeneous distribution across the base metal, the reinforced TiC particles are refined to a 10 µm grain size using SPD and DRX.
2. The enhanced tensile strength and microhardness of the FSP-TiC sample were 482 HV and 832 MPa, respectively, higher than those of FSP-base metal by 41.3% and 39.1%. These results were attributed to superior grain refining, efficient metallurgical bonding, the presence of TiC particle dimples, and the TiC particles impeding impact.
3. The lowest wear rate is efficiently predicted by the suggested regression model, with a confirmation test verifying the error percentile, which falls between ± 4%. This is accomplished by fusing the load-bearing capability and barrier effect of TiC particles with optimal process parameters.
4. Upon morphological analysis of the test parameters, worn surfaces exhibit shallow grooves, delamination, and fine crutches; RSM suggests that sliding velocity and sliding distance are the most crucial variables. The oxide layer shields the surface from wear processes when it builds up at maximum sliding velocity and distance.

Due to the impact and microstructural change of TiC particles, the FSP-TiC sample exhibits better mechanical properties and wear resistance than the FSP-base sample. Additionally, RSM analyses the optimal wear rate of the FSP-TiC sample to determine the most important element. The TiC-reinforced FSP samples could be of interest to companies that produce surgical instruments and bearings. Boiler and storage tank parts can be replaced under the repair and maintenance category in the nuclear and chemical industries.

## Data Availability

The data presented in this study are available through email upon request to the corresponding author.

## Abbreviations

FSP: Friction stir processing
TiC: Titanium carbide
SZ: Stir zone
DRX: Dynamic recrystallization
PAN: Particle-accelerated nucleation
RSM: Response surface methodology
SEM: Scanning electron microscopy
XRD: X-ray diffraction
HV: Vickers hardness
HAGB: High-angle grain boundary
SPD: Severe plastic deformation
PSN: Particle-stimulated nucleation
DOE: Design of experiments
CCD: Central composite design
ANOVA: Analysis of variance
