# Author Correction: A study on microstructural, mechanical properties, and optimization of wear behaviour of friction stir processed AZ31/TiC composites using response surface methodology

**DOI:** 10.1038/s41598-024-73292-0

**Published:** 2024-09-25

**Authors:** T. Satish Kumar, R. Raghu, G. Suganya Priyadharshini, Robert Čep, Kanak Kalita

**Affiliations:** 1 Department of Mechanical Engineering, Amrita School of Engineering, Amrita Vishwa Vidyapeetham, Coimbatore, 641112 India; 2 Department of Mechanical Engineering, Sri Ramakrishna Engineering College, Coimbatore, Tamil Nadu India; 3 Department of Mechanical Engineering, Coimbatore Institute of Technology, Coimbatore, India; 4 Department of Machining, Assembly and Engineering Metrology, Faculty of Mechanical Engineering, VSB-Technical University of Ostrava, 70800 Ostrava, Czech Republic; 5 Department of Mechanical Engineering, Vel Tech Rangarajan Dr. Sagunthala R&D Institute of Science and Technology, Avadi, 600062 India; 6 University Centre for Research & Development, Chandigarh University, Mohali, 140413 India

Correction to: *Scientific Reports*10.1038/s41598-024-69348-w, published online 12 August 2024

The original version of this Article contained an error in Reference 20,

20. Gajević, S., Miladinović, S. & Stojanović, B. Metallic nanocomposites: An introduction. In *Nanotechnology for automotive industry* 265–301 (Elsevier, Amrsterdam, 2022).

now reads:

20. Gajević S., Miladinović S., Stojanović B., Chapter 8—Metallic nanocomposites: An Introduction, Nanotechnology in the Automotive Industry, Micro and Nano Technologies, 155–161. 10.1016/B978-0-323-90524-4.00008-6 (2022).

The original Article has been corrected.

